# Interlinking Antecedent Malaria and Hypertension Through Angiotensin II in India

**DOI:** 10.3389/fcvm.2021.729525

**Published:** 2021-10-11

**Authors:** Aparna Tiwari, Auley De, Veena Pande, Abhinav Sinha

**Affiliations:** ^1^Parasite Host Biology, Indian Council of Medical Research - National Institute of Malaria Research, New Delhi, India; ^2^Department of Biotechnology, Kumaun University, Nainital, India

**Keywords:** malaria, hypertension, evolutionary adaptation, angiotensin II, India

## Two Diseases—Inverse Prevalence Trend in India

**Figure 1:**
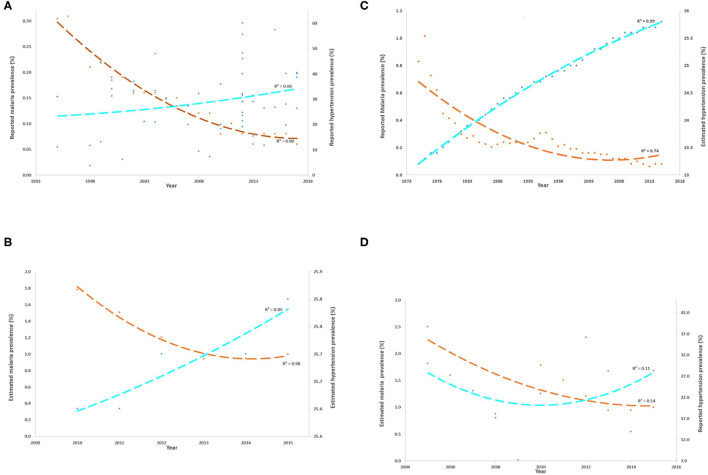
**(A–D)** Inverse trends of reported and estimated burdens of malaria and hypertension in India across various years. The burdens of malaria (Y-axis) and hypertension (secondary Y-axis) are represented as percentage prevalence and the best-fit polynomial trend line (order = 2) is plotted for each, orange dashed line for malaria and fluorescent blue dashed line for hypertension. The calculated *R*^2^ value of the best fit line is also shown. **(A)** shows the reported malaria and hypertension burden in India between 1994 and 2017 as per the published data whereas **(B)** shows the estimated burden of both diseases from 2010 to 2015. The low *R*^2^ value for reported data on hypertension is probably due to the differences in study designs and surveys that reported such data. In addition, because estimated malaria prevalence could not be retrieved beyond 2005 in the past, reported malaria prevalence was also plotted against estimated hypertension prevalence **(C)** and estimated malaria prevalence was plotted against reported hypertension prevalence **(D)**. In all the analyses, it was evident that malaria and hypertension prevalence trends over the years are inversely related in India: the increasing hypertension prevalence is associated with a decreasing prevalence of malaria, although the rate of change is different for both the diseases.

Malaria and hypertension not only differ in their etiopathogenesis but also differ strikingly in their disease burden trend that suggests some links between the two. A quick assimilation and analyses of the available disease burden (reported and estimated) data of both malaria and hypertension show a similar inverse relationship in India as well (Figure 1). As the number of studies/published data on hypertension prevalence in India are scanty, both reported and published studies and nation-wide surveys which reported hypertension prevalence from 1995–2017 (1) and estimated data prevalence of raised systolic blood pressure 1975–2015^1^; were used to plot the burden against the corresponding reported data (1975–2017)^2^; and estimated data 2010–2015; for malaria^3^ (Figures 1A–D). The analyses clearly showed that prevalence of hypertension or raised systolic blood pressure increased over time concomitantly with a corresponding decrease in the burden of malaria in India, thus depicting an inverse trend relationship. This relationship pattern suggests a possible inter-connection between malaria and hypertension, the hypothesized rationale of which is discussed below.

## Proposed Rationale Behind the Inverse Burden

The actual cause of the increasing trend of hypertension prevalence still remains untold in tropical as well as low- and middle-income settings; some scattered evidences do indicate that it could be the result of host evolutionary adaptation to protect against severe/cerebral malaria. Epidemiological studies have reported that the prevalence of hypertension is increasing rapidly in the US population regardless of their socioeconomic status. The reported prevalence of hypertension was higher in blacks as compared to that in whites, in South Asians & Africans as compared to that in Caucasians (2), and in non-Hispanic blacks as compared to that in Hispanic blacks (3). This implies that the black population that has mostly migrated to and settled now in the US and those belonging to South Asian, African and Latin American descent have had a higher odds of developing hypertension and this might be related to their chronic exposure to malaria, that has been rampant in population living in such areas. Studies in areas that are malaria-free since decades, for example UK, also reveal that hypertension is more prevalent among migrants from African and South Asian genetic backgrounds where malaria is endemic (2). What this implies again is that such migrants might be genetically more “at risk” to develop hypertension and malaria could be one of the factors driving such risk. In addition, it is intriguingly observed that hypertension is more prevalent in low-and-middle-income countries (LMIC) compared to high-income countries (HIC) regardless of their socioeconomic setup, again pointing that factors other than the socioeconomic factors, possibly the disease profile of the LMIC, might be putting their population at risk for hypertension. This is also supported by the fact that the population of the African region comprises the highest number in terms of the burden of hypertension compared to the other regions in the world (4). Taking all these evidence together, it might be conceived that malaria, a common element in the population discussed above, might be a contributory factor for increased hypertension risk, although no affirmative evidence currently exists in support of this hypothesis.

The rationale explanation of malaria, as a risk aggravating factor for hypertension, thus explaining this inverse prevalence trend association is rooted in the observation that malaria is believed to confer the strongest evolutionary selection pressure on the human genome among population living in the tropical region resulting in fixation of diverse genetic evolutionary adaptive polymorphisms in the population (5). Genome-wide association studies in malaria-endemic areas have identified several malaria-protective genomic variants in the human genome. Although these polymorphisms tend to provide protection from malaria severity, they also exert their intrinsic detrimental effects on human health in the form of sickle cell disease, α-thalassemia, G6PD deficiency, hemoglobin E disease, and hemoglobin C disease. Natural positive selection of these malaria-protective polymorphisms tend to increase the prevalence of such genetic variants that provide survival advantages to their bearers (6). Moreover, the identified malaria-protective variants may represent just the tip of an iceberg with more variants remain to be still discovered.

In order to be able to attribute malaria as an indirect genetic risk factor for hypertension, it is vital to demonstrate how some of these positively selected malaria-protective genetic variants might increase the risk for raised blood pressure. That will then explain how chronic exposure to severe malaria resulted in a higher prevalence of malaria-protective genetic variants in certain populations and how these genetic variants are associated with an increased risk of hypertension, thus explaining a relatively higher burden of hypertension in areas that are or have been malaria endemic for a long time. This rationale will then also explain how the migrants from these areas, potentially bearing such malaria-protective albeit hypertension-aggravating genetic variants, had a higher prevalence/risk of hypertension as compared to the natives of the region where such population has migrated to.

The proposed rationale may be confounded by a large number of other genetic, constitutional and environmental factors that are still either under investigation or are not yet known to be responsible for contributing to susceptibility to hypertension. For example, it has been shown that the susceptibility to hypertension varies within different ethnic groups living in diverse environmental conditions, thus suggesting that the climate could also be considered as an important selection pressure. It has already been shown that climate adaptation and natural selection play major roles in selecting genetic variants for hypertension (7). In support of this, it has been shown that population living in the tropical regions show a higher prevalence of hypertension susceptible alleles in comparison to those living in cold and dry climate regions (7).

## Proposed Conceivable Hypotheses

Based on the available epidemiological and experimental data, two hypotheses, direct (8) and indirect (9), have been postulated to understand the underlying pathogenic mechanisms that may link malaria exposure to hypertension. The rising trend of hypertension prevalence in low and middle-income countries is backed up by the direct hypothesis which proposes that malaria “directly” contributes to raised blood pressure through chronic inflammation, inflammation-driven angiopoietin-2 mediated arterial stiffness, malaria in pregnancy (leading to hypertensive disorders of pregnancy like gestational hypertension and preeclampsia and low birth weight), and childhood malnutrition in population chronically exposed to malaria (8). A recent cross-sectional population-based study in Côte d'Ivoire compared the risk of hypertension between asymptomatic and symptomatic *Plasmodium* parasitemia and revealed a positive association between symptomatic individuals (malaria) and risk of hypertension (10). This aforementioned co-morbidity scenario shows that acute symptomatic malaria cases have a higher risk of hypertension and thus supports the “direct” hypothesis. Africa has a pool of infectious diseases, especially and predominantly malaria and the rising trend of hypertension prevalence in the African population may be attributed to the chronic inflammation caused by malaria (3, 11). Despite providing some direct explanation to the increasing hypertension burden in areas endemic to malaria, this hypothesis fails to account for the rising trend of hypertension with declining malaria burden, sustained raised blood pressure in areas with currently no malaria, and reasons of hypertension among migrant as their high blood pressure is maintained even after leaving a malaria-endemic area for a generation.

Therefore, the “indirect” hypothesis was put forth which proposed that the elevated blood pressure in the malaria exposed population is the result of certain genetic polymorphisms in the human Renin-Angiotensin System or RAS (*ACE* I/D and *ACE2* rs2106809) which offered protection against severe malaria and hence were naturally positively selected in areas where malaria is/was prevalent. Such positively selected RAS polymorphisms contributed to elevated Angiotensin II levels, that is believed to confer an evolutionary survival advantage in the form of protection from severe malaria in childhood in a variety of ways but is also a direct predictor of raised blood pressure (9). Although this indirect hypothesis explains much of the associations between malaria exposure and augmented risk of hypertension, there are critical knowledge and understanding gaps which are yet to be filled (12).

## Take Home Message

Multifactorial causation of hypertension makes it difficult to attribute malaria as one of the genetic risk factors for hypertension. Identifying the human genetic signatures that can be protected from (severe) malaria and augmenting the risk of hypertension potentially offers a powerful mechanism to understand this complex host-parasite evolutionary relationship. Although the association between RAS polymorphisms and malaria protection has been reiteratively hypothesized, it hasn't been hitherto cohesively investigated, generating strong and good quality evidence, to either accept or refute the proposed hypothesis. Robustly designed molecular epidemiological studies may be the best stepping stones to reveal this unknown niche in different malaria-endemic settings.

## Author Contributions

AT and AD wrote first draft, analyzed the data, and edited the manuscript. AT and AS designed the figure. VP reviewed and edited the manuscript. AS conceived the idea, critically reviewed, edited and finalized the manuscript. All authors read and approved the final manuscript.

## Funding

AT was funded by Council of Scientific and Industrial Research (CSIR), New Delhi [CSIR Award No.: 09/905 (0017)/2017-EMR-I], respectively, for her PhD and AD as Research Associate was funded by Indian Council of Medical Research (ICMR), New Delhi (Award no. fellowship/42/2018-ECD-II).

## Conflict of Interest

The authors declare that the research was conducted in the absence of any commercial or financial relationships that could be construed as a potential conflict of interest.

## Publisher's Note

All claims expressed in this article are solely those of the authors and do not necessarily represent those of their affiliated organizations, or those of the publisher, the editors and the reviewers. Any product that may be evaluated in this article, or claim that may be made by its manufacturer, is not guaranteed or endorsed by the publisher.

## Footnotes

^1^Global health observatory data: raised blood pressure. *WHO Rais blood Press* 2016. Available online at: https://www.who.int/data/gho/data/indicators/indicator-details/GHO/raised-blood-pressure-(sbp-=140-or-dbp-=90)-(age-standardized-estimate).

^2^Magnitude of The Problem, National Vector Borne Disease Control Programme. Available online at: https://nvbdcp.gov.in/index4.php?lang=1&level=0&linkid=420&lid=3699.

^3^*World Malaria Report 2018*. Geneva: World Health Organization (2018). Available online at: https://www.who.int/malaria/publications/world-malaria-report-2018/en/.
